# How Predation and Landscape Fragmentation Affect Vole Population Dynamics

**DOI:** 10.1371/journal.pone.0022834

**Published:** 2011-07-29

**Authors:** Trine Dalkvist, Richard M. Sibly, Chris J. Topping

**Affiliations:** 1 Centre for Integrated Population Ecology (CIPE), Roskilde University, Roskilde, Denmark; 2 Department of Environmental, Social and Spatial Change, Roskilde University, Roskilde, Denmark; 3 Department of Wildlife Ecology and Biodiversity, National Environmental Research Institute, Aarhus University, Rønde, Denmark; 4 School of Animal and Microbial Sciences, University of Reading, Reading, United Kingdom; University of California, Berkeley, United States of America

## Abstract

**Background:**

Microtine species in Fennoscandia display a distinct north-south gradient from regular cycles to stable populations. The gradient has often been attributed to changes in the interactions between microtines and their predators. Although the spatial structure of the environment is known to influence predator-prey dynamics of a wide range of species, it has scarcely been considered in relation to the Fennoscandian gradient. Furthermore, the length of microtine breeding season also displays a north-south gradient. However, little consideration has been given to its role in shaping or generating population cycles. Because these factors covary along the gradient it is difficult to distinguish their effects experimentally in the field. The distinction is here attempted using realistic agent-based modelling.

**Methodology/Principal Findings:**

By using a spatially explicit computer simulation model based on behavioural and ecological data from the field vole (*Microtus agrestis*), we generated a number of repeated time series of vole densities whose mean population size and amplitude were measured. Subsequently, these time series were subjected to statistical autoregressive modelling, to investigate the effects on vole population dynamics of making predators more specialised, of altering the breeding season, and increasing the level of habitat fragmentation. We found that fragmentation as well as the presence of specialist predators are necessary for the occurrence of population cycles. Habitat fragmentation and predator assembly jointly determined cycle length and amplitude. Length of vole breeding season had little impact on the oscillations.

**Significance:**

There is good agreement between our results and the experimental work from Fennoscandia, but our results allow distinction of causation that is hard to unravel in field experiments. We hope our results will help understand the reasons for cycle gradients observed in other areas. Our results clearly demonstrate the importance of landscape fragmentation for population cycling and we recommend that the degree of fragmentation be more fully considered in future analyses of vole dynamics.

## Introduction

Microtine populations in Fennoscandia displays a wide range of population dynamic patterns, shifting along a north-south gradient from persistent multi-annual fluctuations of 3–5 years in the north, to stable populations in the south [1]–[5]. The predominant length of the cyclic period and the amplitude of the multiannual fluctuations both increase toward the north [1], [6]. Analysis of time series data of rodents in Fennoscandia have shown that the latitudinal gradient in microtine dynamics is caused by an underlying cline in the strength of direct density dependence [6]–[8]. Why some microtine populations exhibit multiannually cyclic density fluctuations, while others do not, remains one of the classical problems in ecology (e.g. [2], [5], [9], [10]).

Examination of the multiannual fluctuations has shown that they are a result of a ‘second order’ process [6], that is, they reflect the combined effects of direct and delayed density-dependent processes. The populations are influenced by factors that are a function of the current population density and by factors that are a function of population densities in the past. Direct density-dependent mechanisms tend to stabilise populations, making them less prone to multiannual fluctuations, whereas delayed density-dependent mechanisms do the opposite [11], [12]. Several biological mechanisms are able to produce negative direct density-dependence in rodent populations. One such is crowding leading to competition for space and territories, which has been widely recorded in small mammals [13], [14]. Positive direct density dependence can occur at low population densities, where e.g. mate search becomes more efficient as population densities increase, generating a positive correlation between population density and population growth rate [15]–[17]. Delayed density dependence refers to the time-delayed regulatory effect of past population densities on the reproduction and survival of individuals. It is often interpreted as a sign of trophic interactions, because lagged feedback can readily arise from specialist predator-prey or consumer-resource interactions [18], [19].

A large number of hypotheses have been proposed to explain population cycles and the geographical gradients in density dependence, cycle length and amplitude (for reviews see e.g. [7], [9], [20]). Yet there exists no consensus about what causes these cycles. One of the hypotheses which has received considerable support is the ‘predation hypothesis’, which suggests that the delayed density-dependent effect in the northern populations are generated by a strong numerical response of stationary specialist predators, such as mustelids, which respond to changes in prey densities with a delayed reproductive output [1], [21]. The fluctuations are dampened towards the south by an increased density and diversity of generalist predators [1], [22], [23]. The generalist predators display a functional or migratory response to changes in prey density, and can switch between prey species. The response of these predators to altered prey abundance is nearly instantaneous and does not produce delayed density dependence [1], [3], [22], [24]. Thus if only generalist predators are present, the direct density dependent processes should be sufficient to describe the dynamics [6], [7]. However, the predation hypothesis explicitly incorporates the presence of specialist predators throughout the region. According to the predator hypothesis, specialist predators are thought to cause the fluctuations, whereas generalist predators are considered the cause of the north-south gradient [1], [3], [22], [24], [25]. However recent work suggests that specialist predation may not be necessary for large-scale fluctuations and that these may be generated by other factors depending on their geographical location [26], [27].

The gradient in cycle length and amplitude may also be influenced by landscape heterogeneity. In Fennoscandia, large tracts of continuous habitat dominate northern areas, whereas the south is characterised by a heterogeneous agricultural landscape [28]. Since both predator and prey populations' intraspecific interactions are influenced by landscape heterogeneity [29]–[32], their interspecific interactions are likely to be altered too. Consequently, both direct and delayed density dependence may be affected by habitat fragmentation. This has received attention in some studies [33], [34]. A spatially diverse landscape makes it more difficult for a predator to control its environment and potentially decreases the degree of synchronisation between patches, by allowing prey outbreaks to remain undiscovered by predators [35]–[37]. Accordingly, it can be expected that increasing fragmentation stabilises population densities and decrease the impact of predators on prey populations [34]. However, the degree to which fragmentation alters the dynamics of predators and prey in Fennoscandia is poorly known.

The duration of the rodent breeding season also varies with latitude. For the field vole (*Microtus agrestis*) the length of the breeding season changes from 3–4 months in the north to >7 months in the southern Fennoscandia [38]–[40]. Essentially, seasonality implies that the population dynamics switches between two modes; 1) the summer, or main reproductive period and 2) the winter where no reproduction occurs. The switching between two modes is likely to introduce an inherent oscillator which potentially may be a cause of the multiannual density cycles. Previous studies show that density dependent regulation is strongest during winter [41]–[43] which suggests that the multiannual fluctuations could be influenced by the length of the breeding season [44], [45]. The breeding season hypothesis has gained some support (e.g. [41], [44], [45], [46]), but it is still open whether seasonality is the cause or just a correlate of the cline in population cycling in Fennoscandia.

Investigating the joint effect of predation, fragmentation and breeding season on a large scale in natural systems is inherently difficult. Habitat type, resource availability, species density and species composition of prey and predators covary in Fennoscandia, impeding the separation of explanatory factors in empirical studies. Furthermore, type of predator, breeding season and landscape may be interdependent, since generalist predators are facilitated by an increased diversity of alternative prey, in turn facilitated by a diverse habitat and relative long summer periods [1], [3], [38]. Here we attempt to bridge the gap between the difficulty of obtaining empirical data where predator response, breeding season and landscape heterogeneity are independent, and the need to study these factors separately to understand their impact on prey population dynamics. We investigate and contrast the effects of predation, habitat and breeding season using a realistic agent-based simulation model to examine three descriptive endpoints: mean population size, cycle length and amplitude; and two mechanistic endpoints: direct and delayed density dependence. Agent-based models are particularly useful for this purpose as they allow independent investigation of the impact of single factors by changing one variable at a time [47]–[50]. Their complexity offers the opportunity to incorporate, with a high degree of realism, behavioural plasticity, and individual responses to external perturbations, as well as spatial and temporal landscape change [49], [51]–[54]. They differ fundamentally from models traditionally used to investigate vole cycles in that their aim is to simulate system responses rather than to analytically describe patterns [55]. This means that the resulting data must be subjected to analyses similar to those used for field data, with all the associated complexities and difficulties. However, the advantage is that the experimenter is in total control of the variables in the experiment.

## Materials and Methods

The experiment was designed to investigate the joint effect of predation, fragmentation and breeding season on vole population dynamics. To this end 36 scenarios were designed comprising all possible combinations of three types of predator assembly, four levels of landscape fragmentation, and three durations of breeding season. Each scenario was investigated using the simulation system described below to produce time series for 100 years, with 20 replicates. The sampling was carried out after a ‘burn-in’ period of 100 years.

The ALMaSS system [56] used here is a mature, well tested, comprehensive, but large simulation system; hence detailed model descriptions cannot be presented in text. Full documentation is available in ODdox format [49] from http://www2.dmu.dk/ALMaSS/ODDox/Field_Vole/V1_02/index.html, providing a model overview hyper-linked to a fully documented source code. In addition, ALMaSS is an open source project hosted on the Collaborative Computing Projects site CCPForge (http://ccpforge.cse.rl.ac.uk/gf/), where code and further documentation are hosted. Hence, although an overview is provided below, the reader is directed to the online materials for further details. The model has been tested and found to be able to recreate vole cycle patterns closely similar to those found in a range of real world situations [57]. Appendix S1 provides a description of where to download the source code, together with a link to a zip file with all input files and executable programs used for the simulations. This ensures full replicability of this study.

### Simulation system and landscapes

**Figure 1:**
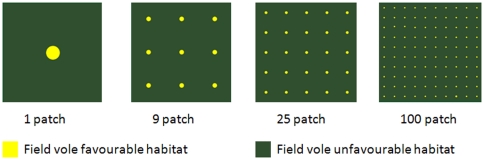
Landscape characteristics of the four 10 km×10 km landscapes. Each landscape comprises two types of habitat: optimal field vole habitat, and unfavourable habitat in which voles cannot feed or breed, but through which they can disperse. Fragmentation was achieved by breaking up the 1.5% optimal habitat into 9, 25 or 100 equally sized patches.

Time series were generated in the general purpose simulation system ALMaSS [56], a spatially explicit agent-based model (ABM) which has been used for a range of applied and theoretical applications (e.g. [47], [58], [59], [60]). ALMaSS is an adaptive system incorporating species specific information on ecology, as well as biotic and abiotic environmental factors. The system models the ecology and behaviour of the field vole at an individual level, together with its interactions with conspecifics, predators and the environment. The time step of the model is one day and has a spatial resolution of 1 meter [56]. The ABM consists of three main parts: the landscape and models of field vole and their predators. A 10×10 km landscape was used comprised of areas of optimal vole habitat, interspersed in a matrix of unsuitable vole habitat. The unsuitable habitat allowed voles to move through freely, but reproduction and long-term survival were restricted to fragments of optimal habitat. Landscape heterogeneity was obtained by fragmenting a one patch homogeneous landscape into 9, 25, and 100 equally sized and spaced patches of optimal habitat (Figure 1). The total area covered with suitable habitat remained unchanged at 1.5% of the total area. Voles could not deplete food resources in the landscape, preventing bottom up regulation from food availability. Density dependence was incorporated through local competition for territories.

### Field voles

The modelled field voles consisted of three life-stages, juveniles and adult females and males. During its life-cycle a vole could engage in a number of behaviours based on information obtained from its local environment and conspecifics. The vole entered the simulation at the location of its mother's nest when it was weaned at day 14 [61], [62]. It entered the simulation as either female or male, assuming an even sex ratio [63] and started off by searching for a suitable territory.

Each day in the simulation the vole would start by assessing the local environment or its territory. Other behaviours could subsequently follow dependent on the information received during this process. A vole needed to have a territory in order to breed. A male could mate with a female if his territory overlapped her position. If this was the case for more than one male, she chose the one closest. Younger voles that found themselves in an older vole's territory of the same gender with an overlap of more than 50% were forced to move. The criteria for assessing territories quality varied with the season and for the mature male during breeding season included assessing for the presence of mature females.

The breeding season started 5^th^ April and ended 1^st^ October [63]–[65]. The length of the breeding season was altered by changing the end date to the 1^st^ September or 1^st^ November to simulate a short or long breeding season respectively. Mortality was modelled as being the result of predation, starvation if they spent too much time in unsuitable habitat, or by reaching their physiological lifespan limit [63]. Mortality also included infanticide attempts if the mature male moved beyond the bounds of his original territory and encountered females with un-weaned young. His success would depend on the age of the young as specified by [66].

### Generalist and specialist predators

The predators were simulated to represent resident mammalian specialist and generalists such as mustelids and foxes, parameter values are given in Table 1. Specialist predators are characterised by a delayed numerical response to changes in prey density [19] and were consequently modelled to require a relatively high number of voles in order to survive and a low number of voles to reproduce. Predator dispersal would occur within a few days of unsuccessful hunting. Their home range and dispersal ability was relatively low in order to represent small mammalian predators.

**Table 1:** Predator parameters and settings.

Predator parameter	Specification	Settings	
		Specialist	Generalist
Reproductive threshold	Number of predated voles needed to produce one offspring. A low value ensures a significant numerical response to high prey density	5	90
Survival threshold	Number of predated voles needed per year to survive. A high value ensure a pronounced decrease in predators in response to low vole density	90	10
Territory size	The predator hunts within its territory and tolerates no overlap with other predators	2500 m^2^	6400 m^2^
Kill efficiency	The probability of killing a vole within the territory. A high value ensures significant pressure on the vole population	9.5%	4%
Failures before dispersal	Number of days without successful predation before dispersal	5 days	20 days
Max dispersal distance	Maximum distance the predator can disperse	500 m	1000 m

Generalist predators on the other hand were modelled with a weakly coupled functional rather than a numerical response and thus required a relatively small number of prey items to survive and a higher number to reproduce. Generalists were relatively unaffected by vole densities and would stay longer in an area with low vole densities before changing to dispersal behaviour. Their home range and dispersal distance were simulated to be greater than specialists to represent the larger generalist mammal predator.

No territory overlap was allowed for predators of the same type. Hunting occurred within the bounds of the territory and voles were predated with the killing efficiency specified in Table 1. Predators reproduced in the spring and mortality were evaluated at the end of the year based on the number of voles consumed (Table 1).

### Data analysis

After a ‘burn in period’ of 100 years mean population size was recorded for each 100 year time series, log_e_ transformed, together with cycle amplitude and length. Amplitude was calculated as maximum/minimum vole population size. Cycle length was estimated from the plot of the autocorrelation function acf() carried out in R 2.12.0 (http://cran.r-project.org/bin/windows/base/old/2.12.0/) by listing the lag at which acf() reached its second positive significant (p<0.05) maximum, while producing stable fluctuations [8], [67], [68]. 95% confidence interval bands (CI) were generated using the formula:
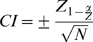
where N is the sample size, z the percent point function of the standard normal distribution and α is the significance level. If acf() was not significantly different from zero or if irregular fluctuations were present, then we concluded that no stable periodicity existed for the analysed time series and the cycle length was recorded as zero. The generated time series were subsequently analysed using standard second-order autoregressive analyses [67], [68] to determine the coefficients of direct (AR1) and delayed (AR2) density dependence. These analyses were performed using R 2.12.0, analyses of variance used Minitab 15.

## Results

**Figure 2:**
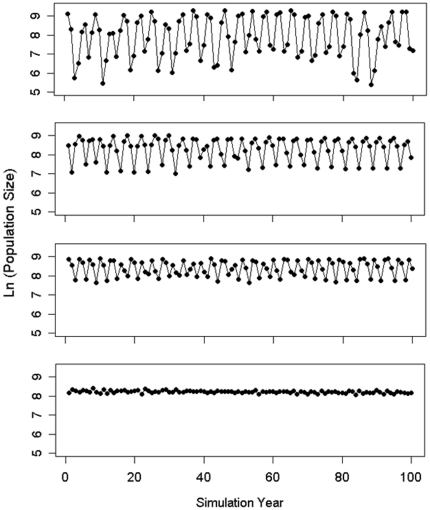
Examples of time series of vole density (log10 transformed). The graph displays field vole population size in landscapes containing a specialist predator but differing in degree of fragmentation. Landscape fragmentation increases from the top (1 patch) to the bottom (100 patches).

**Figure 3:**
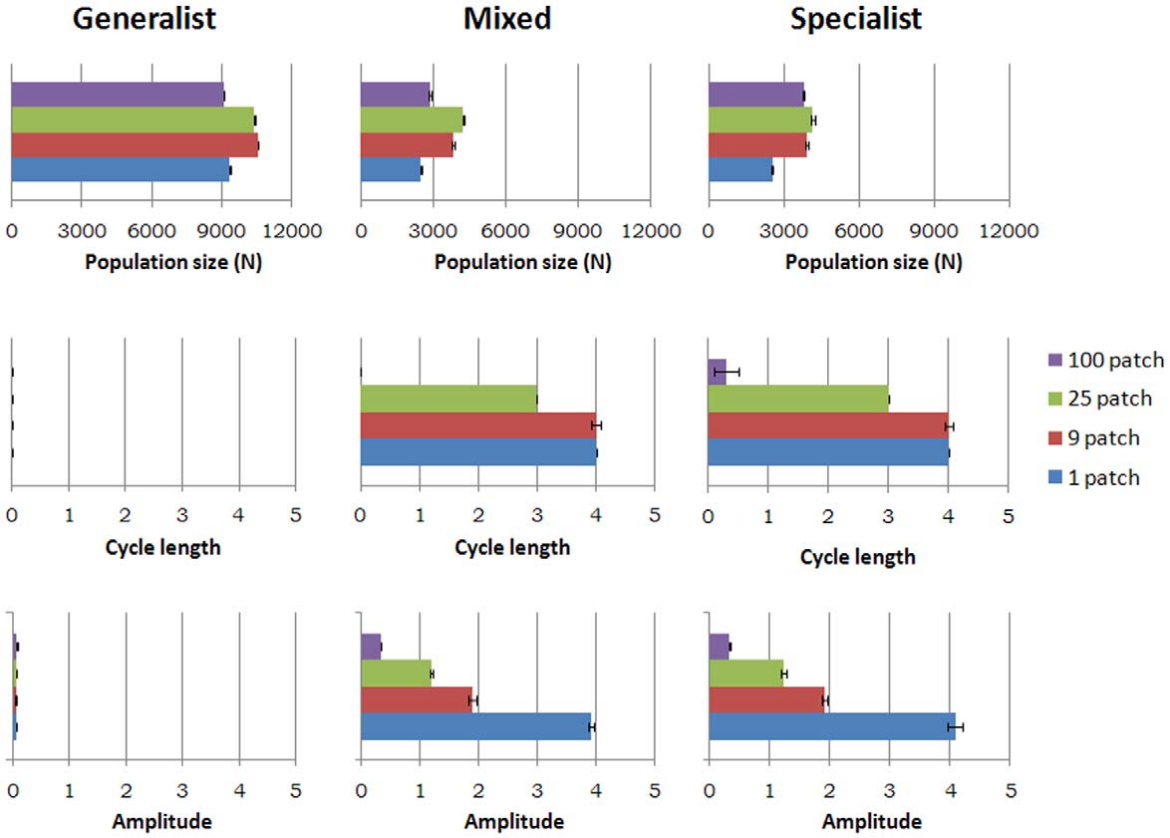
Mean population size, cycle length and amplitude, and mean values for the intermediate vole breeding season. Each column refers to one of the three types of predators as indicated at the top of the figure. The colour code for each graph refers to the level of heterogeneity in the landscape as shown in the key at the right. Bars indicate standard errors.

Model vole population dynamics exhibited a range of patterns from four year cycles to stable populations (Figure 2 and 3). Landscape structure, the type of predator and the interaction between the two had marked effects on all measured parameters in the analyses of variance, whereas the effects of length of breeding season and its interactions were minor (Table 2). We therefore focus here on landscape structure, type of predator and their interactions.

**Table 2:** Analysis of variance.

		R_adj_^2^ (%)				
		Descriptive variables			Mechanistic variables	
	DF	N	CL	Amp	AR1	AR2
Source						
Land (L)	3	34	21	7	33	22
PrType (Pr)	2	30	59	82	22	55
BSeason (B)	2	0	0	2	5	0
L*Pr	6	17	14	2	24	15
L*B	6	0	1	2	1	1
Pr*B	4	0	1	1	3	0
L*Pr*B	12	0	2	1	3	1

Percentage of variation accounted for (R_adj_^2^) in an analysis of variance of the effects of landscape structure (L), predator type (Pr), and Breeding season (B) on the descriptive variables: population size (N), amplitude (Amp) and cycle length (CL), and on the mechanistic variables: direct density dependence (AR1), and delayed density dependence (AR2). The * illustrates the interaction between the listed parameters. All effects were statistically significant (p<0.05).

Analyses of variance showed that mean vole population size density was mainly affected by landscape structure and predator assembly with the two factors accounting for similar amounts of the total explained variance (∼30%), whereas the interaction between the two explained 17% (Table 2). For all predator assemblages, increasing habitat fragmentation increased mean vole population size up to 25 patches after which a reduction in population size occurred (Figure 3). Introducing specialist predators, whether or not generalists were present, more than halved vole population sizes.

Cycle length and amplitude were largely determined by the predator assembly, which described between 59–82% of the total explained variance (Table 2). Populations did not cycle in the 100-patch landscapes, or if exposed only to generalist predators, but in all other cases cycles occurred (Figure 3). As the landscape progressively became more homogeneous, cycle length and amplitude increased.

**Figure 4:**
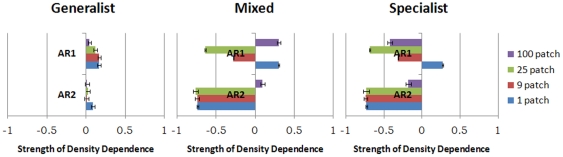
Direct and delayed density dependence, measured by AR1 and AR2 respectively. Conventions as in Figure 3.

Direct density dependence, AR1, was most affected by landscape structure, followed by the landscape*predator interaction, and lastly the predator assembly (Table 2). With generalist predators AR1 was weakly positive for all fragmentation levels (Figure 4). With specialist predators AR1 shifted from positive to negative as fragmentation increased. The response was similar with mixed predators except that with 100 patches AR1 became positive.

Delayed density dependence, AR2, was most affected by predator assembly (55%), followed by the landscape structure (22%) and then their interaction (15%) (Table 2). No delayed density dependence was observed when voles were exposed only to generalist predators (Figure 4). Introducing specialist predators made AR2 strongly negative, below −0.7, except in the 100 patch landscape, where it was weakly negative for specialist and weakly positive for mixed predators.

## Discussion

The existence of population cycles is best judged by their stable multiannual fluctuations and amplitude, and as expected this was associated with delayed density dependence. Cycles were absent if the only predators were generalists, or if the landscape was fragmented into 100 patches (Figure 3). There is good agreement between our results and those obtained by workers in Fennoscandia. Thus in the North, where the landscape is relatively homogeneous and specialist predators are abundant, there are pronounced population cycles with associated high delayed density dependence (i.e., strongly negative AR2), and overall vole mean population size are relative low. As we move towards the south and fragmentation level and generalist abundance increases, cycle length and direct density dependence decreases, while delayed density dependence remains stable. In the South where landscapes are fragmented and generalist predators are abundant, there are no cycles and no delayed density dependence, and mean vole population size are higher. In Fennoscandia predator type and landscape fragmentation covary, so their effects are confounded. This ambiguity is here resolved by modelling, which has allowed us to distinguish the effects of predator type and landscape fragmentation.

Population cycles only occurred in our simulations in fairly homogeneous environments containing specialist predators. To an extent this concurs with previous interpretations, which have usually considered predation the main factor driving the population dynamics of Fennoscandian microtines [1], [3], [22], [24]. In the past fragmentation has received less attention (but see [33], [34], [69]). However our results suggest that low fragmentation levels as well as the presence of specialist predators are necessary for the occurrence of population cycles. This is not surprising because ecological processes influence and are influenced by the landscape [29]–[31], [51], so predator-prey dynamics are likely to be affected by landscape structure as well as by the predator assembly.

One perhaps unexpected result was that intermediate fragmentation levels increased the number of voles. Subsequent analysis to test this pattern showed that a predator in the homogeneous landscape experienced few days during the year without successful predation. Therefore it had a relatively constant supply of voles and could remain stationary for longer and thus kill a large proportion of voles. As fragmentation levels increased up to 25 patches the predator experienced around 18% extra days without successful predation and a 23% lower predation rate. Consequently, the predators' regulatory effect decreased. By contrast, predators in fragmented landscapes had an increased risk of driving voles to local extinction as the habitat size became smaller [29], [32], [70]. Subsequent analyses showed there to be around 60–70% unoccupied patches in the most fragmented landscape. This is why mean vole population size decreased in the most heterogeneous landscape.

Predator dispersal in fragmented landscapes also accounts for the reduction in delayed density dependence that occurred there. Predators in heterogeneous landscapes were more often forced to disperse by lack of food, and this diluted their effects on population dynamics. Similar effects of fragmentation are seen in other systems [35], [71]. The extent to which fragmentation reduces predator impact depends on whether they are specialist or generalist. Specialists may retain some impact because they occasionally occur at high population sizes [1], [25]. On the other hand populations exposed to generalist predators displayed very low levels of delayed density dependence (Figure 4). This is because generalists responded near instantaneously to changes in prey population size without affecting their abundance [72], [73].

The overshadowing of predator effects by fragmentation may in part explain the difficulty of reconciling vole time-series from Britain [26] with those from Fennoscandia, and the difficulty experienced by Lima et al. [74] in explaining differences in vole dynamics along similar latitudinal gradients in Fennoscandia and Russia. It would be interesting to identify the precise variations in the predator complex and the degree of fragmentation in these gradients, to see if they match our predictions.

Negative direct density dependence results from direct competition for food or territories, and is indicated by negative values of AR1. We found no negative direct density dependence in the absence of specialist predators or in homogeneous environments (Figure 4). This lack of intraspecific competition was a result of populations being kept below potential carrying capacity [75], [76], which was more effective in homogeneous environments and/or when predators were generalists, as we have illustrated. Field studies have shown that density dependent regulation is strongest during winter [41]–[43] which suggests that multiannual fluctuations could be influenced by the length of the breeding season, but in our analysis the latter had little effect. However, other related factors such as changes in predation efficiency due to snow cover, and vole food limitation during winter were not investigated in this study. Further data addressing these issues would be necessary before eliminating the length of breeding season as an important factor in shaping the multiannual fluctuations. Our results clearly demonstrate that landscape fragmentation can produce the increased strength in negative direct density dependence observed in the Fennoscandian gradient as often has been assigned to the increased abundance of generalist predators and we stress that landscape structure should receive more consideration when analysing multiannual fluctuations.

### Conclusion

In agreement with the literature, specialist predators generated delayed density dependence and vole population cycles, whilst fragmentation and generalist predators dampened these effects. Interaction effects were surprisingly strong, suggesting that voles in different landscapes under the same predator assemblage could have distinctly different population dynamics, depending on the level of landscape fragmentation. The length of the vole breeding season had few effects. Naturally, as in the real world, our results are system-configuration dependent, but they indicate that the impact of fragmentation should be considered to a greater degree when analysing vole cycles.

## Supporting Information

Appendix S1 **Assessing ALMaSS code on CCPForge.**
(DOC)
