# Supplementary material for: How Predation and Landscape Fragmentation Affect Vole Population Dynamics
Source: PLoS One. 2011 Jul 29;6(7):e22834. doi: 10.1371/journal.pone.0022834 (PMC3146495; doi:10.1371/journal.pone.0022834)
Supplement: Appendix S1 — Assessing ALMaSS code on CCPForge. (DOC) [file pone.0022834.s001.doc]

## Appendix S1: Assessing ALMaSS code on CCPForge

### Downloading the source code

The ALMaSS source code can be downloaded from CCPForge page following the link <http://ccpforge.cse.rl.ac.uk/gf/project/almass/docman/?subdir=159>. It is provided as a zip file with the name SourceCode.zip. It can be made compatible with 32 bit computers as well as Linux systems by not defining __64BIT and by defining __UNIX in almass_setup.h respectively. The default is 64 bit and Windows system. This allows compiling ALMaSS using compilers such as Microsoft Visual Studio or GCC. We have used Boost libraries version 1.38.0 and Microsoft Visual Studio to create the executable ALMaSS program for 64 bit machines. The length of the breeding season is specified in the file ALMaSSDefines.h by un-ticking the desired length and placing // in front of the two other options. The defines for long, normal and short breeding season are __LONG_BSEASON, __NORMAL_BSEASON, and __SHORT_BSEASON respectively. They are defined by removing // in front of #define. One of these defines has to be selected for the simulation to run.

### Input files

The input files needed for running ALMaSS can be found in the folder <http://ccpforge.cse.rl.ac.uk/gf/project/almass/docman/?subdir=159> with the name InputFiles_Programs.zip. This zip file also contains a readme.txt file describing how to run the program, how to change between scenarios and how to alter the number of replicates. The executable programs we have used for running the simulations are also included in this folder which allows those interested to run the programs on 64 bit Windows machines.

### Publishing feedback

If interested, critics, questions or tributes can be posted in the Forums tap if you join the project. This can be acquired by following the link <http://ccpforge.cse.rl.ac.uk/gf/project/almass/> and press ‘Request to join project on the right hand side of the page. Additional help can also be provided if needed after joining the project by listing the question under the help tab in Forums.
